# Collagen type I alpha 2 acts as a potential diagnostic biomarker and therapeutic targets for the prognosis in gastric cancer

**DOI:** 10.1007/s12672-026-04924-2

**Published:** 2026-03-31

**Authors:** Jingjing Dai, Xudong Song, Abdusemer Reyimu, Zihao Gao, Chenggong Zhang, Fan Ding, Dezhu Dai, Liang Shi, Xiao Han, Wubi Zhou, Guoquan Tao

**Affiliations:** 1 Department of Medical Laboratory, The Affiliated Huai’an No.1 People’s Hospital of Nanjing Medical University, Huai’an, 223300 Jiangsu People’s Republic of China; 2 Department of Gastrointestinal Surgery, The Affiliated Huai’an No.1 People’s Hospital of Nanjing Medical University, Huai’an, 223300 Jiangsu People’s Republic of China; 3 Department of Pathology, The Affiliated Huai’an No.1 People’s Hospital of Nanjing Medical University, Huai’an, 223300 Jiangsu People’s Republic of China

**Keywords:** Gastric cancer, Bioinformatics analysis, Prognosis, COL1A2, Clinical parameters

## Abstract

**Background:**

Gastric cancer (GC) is a common digestive tract cancer whose high heterogeneity and invasiveness lead to a low survival rate. Therefore, it is necessary to explore the potential molecular mechanism of GC.

**Methods:**

Three genes and one miRNA expression microarray dataset were downloaded from the GEO database. The gene expression profiles of the cancer group and normal group were compared in each dataset, and differentially expressed genes (DEGs) and miRNAs were identified with GEO2R. GO enrichment and KEGG pathway analysis of DEGs were performed with the R package clusterProfiler. The interaction network of DEGs was visualized with Cytoscape, and clusters and key genes were identified. According to the key DEGs, a prognostic risk model of GC was established by Cox regression. The patients were subdivided into high-risk and low-risk groups based on the median value of the risk score, and the model performance was evaluated by ROC curve and survival analyses. The risk model was combined with clinicopathological features to establish a nomogram, and a calibration chart was used to evaluate the prediction accuracy of the nomogram. The ROC curve was drawn to predict the ability of genes to differentiate tumour tissues from normal tissues. The starBase database was used to construct a regulatory network consistent with the miRNA–mRNA hypothesis. The expression of COL1A2 was analysed by immunohistochemistry/immunocytochemistry (IHC/ICC) in 150 patients with gastric cancer and a GC cell line. The correlations between COL1A2 expression and clinical features were analysed.

**Results:**

A total of 106 DEGs and 113 differentially expressed miRNAs were identified from the gastric cancer dataset. PPI network screening revealed the two most significant modules and identified the dominant gene. A prognostic risk model composed of six prognostic genes (COL1A2, COL1A1, COL3A1, SPARC, LUM and BGN) was constructed by Cox regression analysis. The prognosis of the high-risk group was poor (*P* < 0.001). ROC curve analysis (AUC = 0.732) showed that the model better predicted the 5-year survival rate of patients. In addition, the six prognostic genes had appropriate diagnostic ability for GC. A potential ceRNA network of model genes in GC was constructed through the starBase database. IHC showed that COL1A2 was highly expressed in gastric cancer and GC cells. COL1A2 expression was significantly correlated with lymph node metastasis.

**Conclusion:**

This study reveals the potential biomarkers and related pathways of GC and provides a theoretical basis for the diagnosis and prognosis prediction of GC.

**Supplementary Information:**

The online version contains supplementary material available at 10.1007/s12672-026-04924-2.

## Introduction

Gastric cancer (GC), one of the most common malignancies, is a diverse and complex disease [1]. East Asia has the highest incidence of gastric cancer, followed by Eastern Europe and Central Europe. Patients from Africa are often younger and more likely to be female [2] than other patients. Although the combination of surgery, radiation, chemotherapy, and targeted therapy increases the survival duration of patients with GC, the mortality of GC is still high, and patients with GC still have a poor prognosis [3]. To date, the specific molecular pathological mechanism and diagnostic markers are still unclear. Therefore, it is necessary to search for novel sensitive and specific prognostic markers for gastric cancer.

The genes in the cancer tissues of clinical gastric cancer patients contain numerous mutations [4]. Thus far, gene chip sequencing technology has been used to assess tumour cells and has revealed that there are extensive gene sequence changes [5]. These factors lead to abnormal expression of proteins, detection of which is playing an increasingly important role in the early diagnosis and treatment of gastric cancer [6]. Thus far, some genes and proteins have been used for the diagnosis of gastric cancer, such as HER2, KI-67, and EGFR. Activation of HER2 can lead to tumour progression, which has led to research on the use of HER2-targeted drugs in cancer with HER2 gene alterations. This has been demonstrated in the context of HER2 gene amplification in gastric cancer [7]. For gastric cancer with abnormal HER2 expression, drugs targeting HER2 are available and have become part of the treatment plan. It is precisely this molecular diversity and often the lack of common carcinogenic driving mutations that lead to the adverse treatment reactions that patients often face when undergoing treatment for gastric cancer. Therefore, there is an urgent need to widely identify the genomic abnormalities of GC and elucidate the molecular basis of GC to improve early diagnostic evaluation and reduce the mortality rate of GC.

The recent development of the combination of bioinformatics and chip sequencing technology has provided technical experimental methods for the mining and discovery of GC diagnostic genes [8]. However, integrating numerous tumour markers to improve the diagnosis and prognostic analysis of GC tumours remains a clinical challenge. Therefore, in this experiment, GC gene data from a gene database were integrated with clinical data to construct a prognostic model and search for biomarkers directly related to prognosis, providing a theoretical basis for the diagnosis and prognosis evaluation of GC.

## Materials and methods

### Gene expression profile data collection

A microarray dataset of gastric cancer was obtained from the GEO database (http://www.ncbi.nlm.nih.gov/geo/). The selection criteria for the expression profile were as follows: (1) the test sample was tissue, (2) all tissues were diagnosed as gastric cancer tissues and normal tissues, (3) the gene expression profile was for mRNA, (4) the number of samples was sufficient (50 or more samples), (5) detection probes were able to identify corresponding genes and convert probe signals into corresponding gene expression symbols, and (6) the analysis information was complete. Three mRNA expression datasets (GSE29272, GSE33335 and GSE63089) were selected. The GSE29272 dataset is based on the GPL96 Platform (Affymetrix Human Genome U133A array). The GSE33335 and GSE63089 datasets are based on the GPL5175 Platform (Affymetrix Human Exon 1.0 St array). The GSE93415 dataset is based on the GPL19071 Platform (Exiqon miRCURY LNA microRNA array). GSE29272 contained 134 GC tissues and 134 adjacent normal tissues, GSE33335 contained 25 GC tissues and 25 adjacent noncancerous tissues, and GSE63089 contained 45 GC tissues and 45 adjacent normal tissues. In addition, we randomly selected the GC gene chip dataset GSE15459 in this study to validate the risk model. The GSE15459 dataset contains gene expression data and survival information for 192 GC patients. The details of the microarray are shown in Table 1.

**Table 1:** Gene expression dataset characteristics

Dataset	Platform	Number of samples	Region	Survival outcome
GSE29272	Affymetrix Human Genome U133A array	268	USA	None
GSE33335	Affymetrix Human Exon 1.0 St array	50	China	None
GSE63089	Affymetrix Human Exon 1.0 St array	90	China	None
GSE15459	Affymetrix Human Genome U133 Plus 2.0 Array	192	Singapore	Overall survival

### Data processing and screening of DEGs

The online analysis tool GEO2R (https://www.ncbi.nlm.nih.gov/geo/geo2r/) was used to analyse the DEGs of gastric cancer and its adjacent tissues. GEO2R is a dataset analysis tool based on the R language program that can compare two groups of samples under the same experimental conditions and identify DEGs.

The upward and downward trends in gene expression were distinguished by the fold change (FC) value. The screening criteria set in this study were *P* < 0.05 and |logFC|> 1 [9] (screening DEGs with expression differences of more than 2 times).

### GO functional annotation and KEGG enrichment analysis

The "clusterProfiler" package of R software (Version 3.6.3) was used for GO enrichment analysis and KEGG signal pathway analysis of DEGs [10, 11, 15]. The screening criteria were DEGs associated with a single term, and the DEGs were arranged according to the enrichment degree. *P* < 0.05 was considered to indicate statistical significance. The GO database was used to analyse the functional enrichment in the biological process (BP), cellular component (CC) and molecular function (MF) categories.

### Construction of the PPI network and screening of central genes

The STRING11.0 online database (https://www.string-db.org/) was used to explore the protein‒protein interaction (PPI) network among DEGs [12]. The PPI network was constructed by using Cytoscape software (Version 3.8.2, http://www.cytoscape.org/), and the network topology was analysed. The plug-in MCODE was used to analyse the PPI network module [13]. An MCODE score > 5 was used as the screening criterion for significant modules, and a number of genes > 5 was used as the subnetwork. The MCC and Degree algorithms were used to select the top 10 genes [14], and the overlapping genes were considered central genes.

### Validation of key prognostic genes in the TCGA database

RNA SEQ data related to gastric cancer were retrieved from the TCGA database (https://portal.gdc.cancer.gov/). RNA SEQ data of 375 groups of gastric cancer tissues and 32 groups of normal tissues were obtained. At the same time, the clinical information of patients corresponding to the source of gastric cancer samples was also obtained. Among them, 367 patients with gastric cancer had survival time and survival status information. The gene expression data were sorted and standardized by the R software (Version 3.6.3) “limma” package [10]. Differences in the expression of key genes in tumour and normal tissues were analysed by t test and with GraphPad Prism 5.0 software.

### Construction and effectiveness evaluation of the prognostic risk model

Univariate Cox regression analysis was used to screen the genes related to prognosis, and the indices with *P* < 0.05 were selected for further analysis. Multivariate Cox regression analysis was used to construct the prognostic risk model of gastric cancer. The risk score of each patient was calculated according to the model formula, and the patients were divided into high-risk and low-risk groups according to the median score. The "pheatmap" package in R software (Version 3.6.3) [10] was used to draw the expression trend heatmap of the six model genes. Kaplan‒Meier (KM) survival analysis was used to compare the prognoses of the high-risk and low-risk groups. In addition, the prediction ability of the model was evaluated by drawing the receiver operating characteristic (ROC) curve and calculating the area under the curve (AUC). The nomogram model of the risk score and clinicopathological data related to prognosis was constructed by using the R software (Version 3.6.3) “rms” package [10]. The closer the calibration curve was to 45°, the better the prediction ability of the nomogram.

### Prediction of the miRNA‒mRNA network

Increasing evidence shows that the miRNA‒mRNA regulatory mechanism exists widely in cancer. Therefore, we sought to explore the mechanism of miRNA‒mRNA regulation of model genes. The target miRNAs of the model gene mRNAs were predicted by the starBase database (https://starbase.sysu.edu.cn/) and analysed by seven prediction programs (PITA, miRanda, DIANA microT, PicTar, miRmap, RNA22 and TargetScan). The target miRNA screening conditions were as follows: programNum >  = 3, CLIP-Data >  = 3, pan-Cancer >  = 1. In addition, we further analysed the correlations between target miRNAs and mRNAs and screened for miRNAs that were most suitable for the ceRNA hypothesis. By comprehensively analysing miRNA‒mRNA interactions, the miRNA‒mRNA network of STAD was established.

### Tissue samples

In this study, 150 pairs of gastric adenocarcinoma and adjacent normal tissues were collected from the Affiliated Huaian No. 1 People’s Hospital of Nanjing Medical University from 2016 to 2018, including 106 males and 44 females. There were 75 cases with lymph node metastasis and 75 cases without lymph node metastasis. The diagnosis was made by two senior pathologists. All cases were confirmed by histopathology, patients did not receive any preoperative radiotherapy or chemotherapy, clinical data were complete, and informed consent forms were signed.

### Immunohistochemical staining

Paraffin slices were dewaxed in xylene and hydrated with gradient alcohol, and then the slices were placed in antigen repair solution. After 15 min of microwave antigen repair, the slices were washed with PBS buffer and sealed with 5% BSA for 30 min. After the sealing solution was removed by filter paper, the first antibody (COL1A2, Abcam, ab96723, 1:300 dilution) was added, and then the slices were placed in a 4 °C refrigerator for 8 h. After washing with PBS buffer, the secondary antibody (goat anti-rabbit IgG H&L (HRP), Abcam, ab6721, 1:500 dilution) was added, and the slices were incubated at room temperature for 1 h. The slices were rinsed with PBS buffer and treated with DAB for 10 min, and then the slices were dehydrated and sealed. Finally, the immunohistochemical results of the slices were observed through an electron microscope. The criteria for immunohistochemical staining results were as follows: H-score: staining intensity × positive cell rate. It was double-blinded by two pathologists.

### Cell culture and immunohistochemical staining

Gastric cancer cell lines (AGS, MKN-28, MKN-45) and normal gastric epithelial cell lines (GES-1) were donated by the Chinese Center for Disease Control and Prevention. The cell lines were cultured in DMEM containing 10% (volume fraction) foetal bovine serum (FBS) and placed in a cell incubator containing 5% (volume fraction) CO2 at 37 °C. Cells in logarithmic growth phase were digested with 0.25% trypsin and resuspended in complete medium. The cells were seeded on glass slides and cultured in an incubator at 37 °C under 5% CO2 and > 90% humidity for 24 h. The slide or cover glass was removed and immersed in PBS (pH = 7.4) 3 times. After that, the cells were immersed in 4% paraformaldehyde for 20 min for fixation, washed with PBS 3 times, and sealed with 1% BSA for 20 min. The cells were incubated overnight at 4 °C with the primary antibody (COL1A2, Abcam, ab96723, 1:500 dilution), washed with PBS three times, and then incubated with an appropriate amount of secondary antibody (goat anti-rabbit IgG H&L (HRP), Abcam, ab6721, 1:1000 dilution, 37 °C, 20 min). After washing with PBS 3 times, DAB was used for 5 min, gradient alcohol was used for dehydration, and the sealing sheet was made transparent with xylene. Finally, the immunohistochemical results of the slices were observed through an electron microscope.

### Statistical processing

All statistical analyses were performed using R software (Version 3.6.3, www.r-project.org), SPSS 17.0 and GraphPad Prism 5.0 software. For comparison, two-tailed Student’s t test and Welch’s t test were used as appropriate. Univariate and multivariate Cox regression analyses were performed to evaluate the survival rate. ROC curves were used to evaluate the ability of prognostic factors to predict the prognosis of patients and the ability of genes to diagnose cancer.

## Results

### Identification of DEGs

**Fig. 1:**
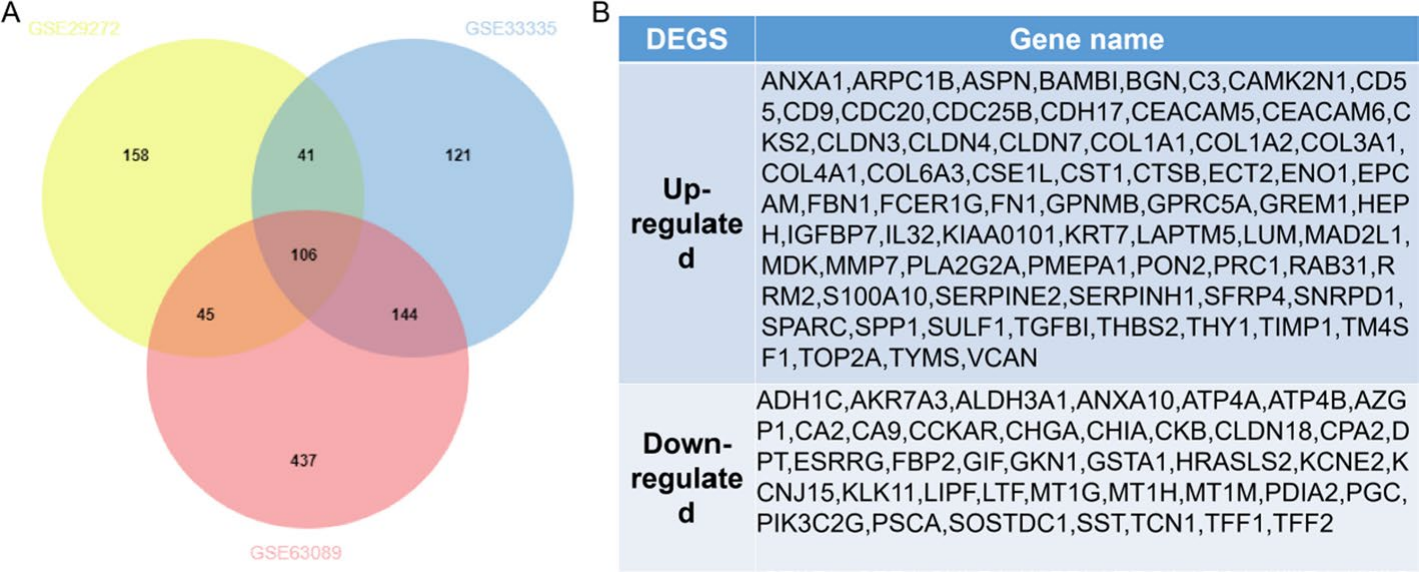
Identification of DEGs in Gastric Cancer (GC). **A** Venn map of DEGs in GSE29272, GSE33335 and GSE63089. **B** Up- and downregulated expression of 106 DEGs in tumour tissues. DEGs: differentially expressed genes

Using the online analysis tool GEO2R, 350, 412 and 732 DEGs were identified through screening of three GC datasets (GSE29272, GSE33335 and GSE63089). The Wayne map shows that there were 106 overlapping DEGs (Fig. 1A), including 67 upregulated genes and 39 downregulated genes (Fig. 1B).

### GO function and KEGG enrichment analysis

**Fig. 2:**
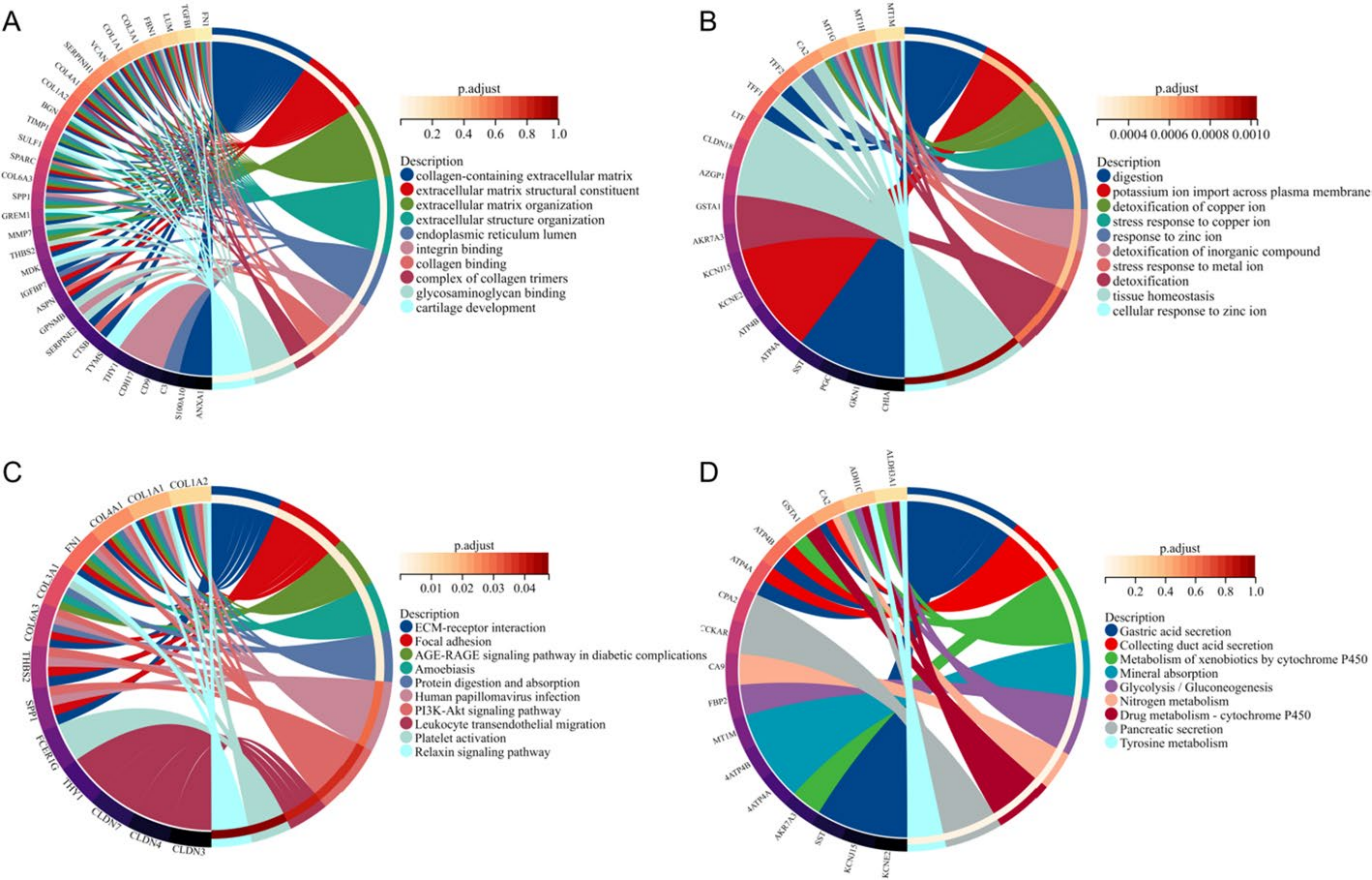
Functional enrichment of DEGs. **A** Gene Ontology (GO) analysis showed the biological functions of upregulated genes. **B** GO analysis showed the biological functions of downregulated genes. **C** Kyoto Encyclopedia of Genes and Genomes (KEGG) analysis showed the signalling pathways of upregulated genes. **D** KEGG analysis showed the signalling pathways of downregulated genes. Permission has been obtained from Kanehisa Laboratories for using the KEGG pathway database [15]

Using the clusterProfiler package, GO function annotation and KEGG pathway enrichment analysis were performed on 106 DEGs, and the top 10 significant analytical results were taken. The GO functional annotation results showed that the upregulated DEGs were associated mainly with collagen-containing extracellular matrix, extracellular matrix structural constituent, extracellular matrix organization, endoplasmic reticulum lumen, integrin binding, collagen binding, complex of collagen trimers, glycosaminoglycan binding, and cartilage development (Fig. 2A). The GO functional annotation results showed that the downregulated DEGs mainly involved digestion, potassium ion import across the plasma membrane, detoxification of copper ions, the stress response to copper ions, the response to zinc ions, detoxification of inorganic compounds, the stress response to metal ions, detoxification, tissue homeostasis, and the cellular response to zinc ions (Fig. 2B). KEGG pathway analysis showed that the upregulated DEGs were enriched mainly in ECM–receptor interaction, focal adhesion, AGE-RAGE signalling pathways in diabetic complications, amoebiasis, protein digestion and absorption, human papillomavirus infection, the PI3K-Akt signalling pathway, leukocyte transendothelial migration, platelet activation, and the relaxin signalling pathway (Fig. 2C). KEGG pathway analysis showed that the downregulated DEGs were enriched mainly in gastric acid secretion, collecting duct acid secretion, metabolism of xenobiotics by cytochrome P450, mineral absorption, glycolysis/gluconeogenesis, nitrogen metabolism, drug metabolism—cytochrome P450, pancreatic secretion, and tyrosine metabolism (Fig. 2D).

### PPI network construction and key cluster analysis

**Fig. 3:**
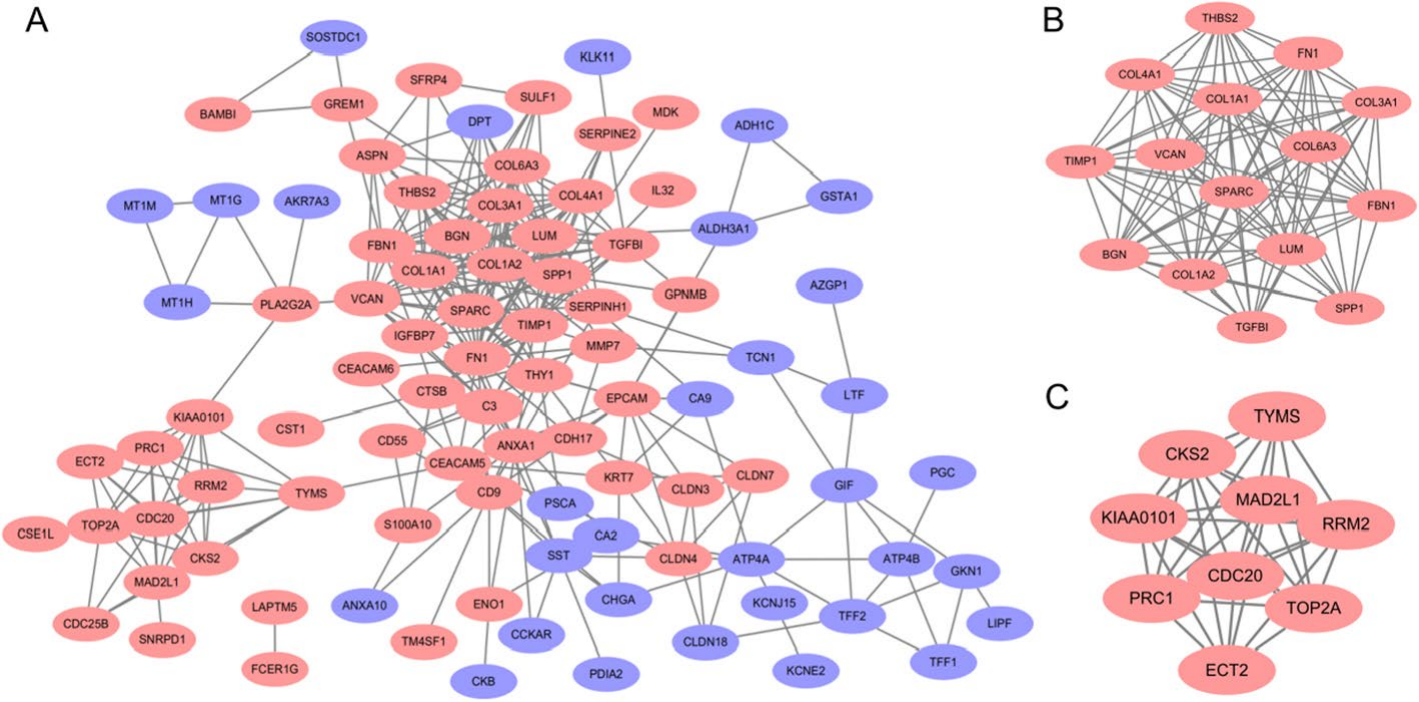
Visualization of the protein–protein interactions (PPI) of DEGs derived from STRING using Cytoscape software (Version 3.8.2, http://www.cytoscape.org/). Upregulated DEGs are shown as red dots, and downregulated DEGs are shown as purple dots. **A** In all, 93 nodes and 317 edges are displayed. **B**, **C** Cluster 1 and Cluster 2 were determined by the MCODE plug-in in Cytoscape software. Circles represent genes, and lines between genes represent interactions between genes. DEGs: differentially expressed genes. MCODE: molecular complex detection

The PPI network of DEGs composed of 93 nodes and 317 connections was constructed with the STRING online database, and the confidence score was more than 0.4. The visualization of the PPI network was carried out with Cytoscape software (Version 3.8.2) (Fig. 3A). Through the MCODE plug-in, the parameters were set to degree cut off = 2, node score cut off = 2, k-core = 2, and max depth = 100 to obtain 6 clusters; the first two clusters had scores of 13.571 and 8.750 (Figs. 3B and 3C). In the cytoHubba plug-in of Cytoscape, the MCC and Degree algorithms were selected to screen for the top ten genes, and the overlapping genes of the two algorithms were regarded as the key genes. Nine key genes, COL3A1, FN1, BGN, SPARC, COL1A1, COL1A2, FBN1, LUM and TIMP1, were identified (Table 2).

**Table 2:** Top 10 central genes of maximal clique centrality (MCC) and degree scores in the protein‒protein interaction (PPI) network

Genes	Expression	MCC	Genes	Expression	Degree
COL3A1	Up	1.54E+07	FN1	Up	28
FN1	Up	1.54E+07	COL3A1	Up	21
BGN	Up	1.54E+07	COL1A1	Up	21
SPARC	Up	1.54E+07	TIMP1	Up	21
COL1A1	Up	1.53E+07	BGN	Up	19
COL1A2	Up	1.53E+07	COL1A2	Up	19
FBN1	Up	1.46E+07	SPP1	Up	18
THBS2	Up	1.45E+07	SPARC	Up	17
LUM	Up	1.17E+07	FBN1	Up	17
TIMP1	Up	1.14E+07	LUM	Up	17

### Expression of key genes in gastric cancer

**Fig. 4:**
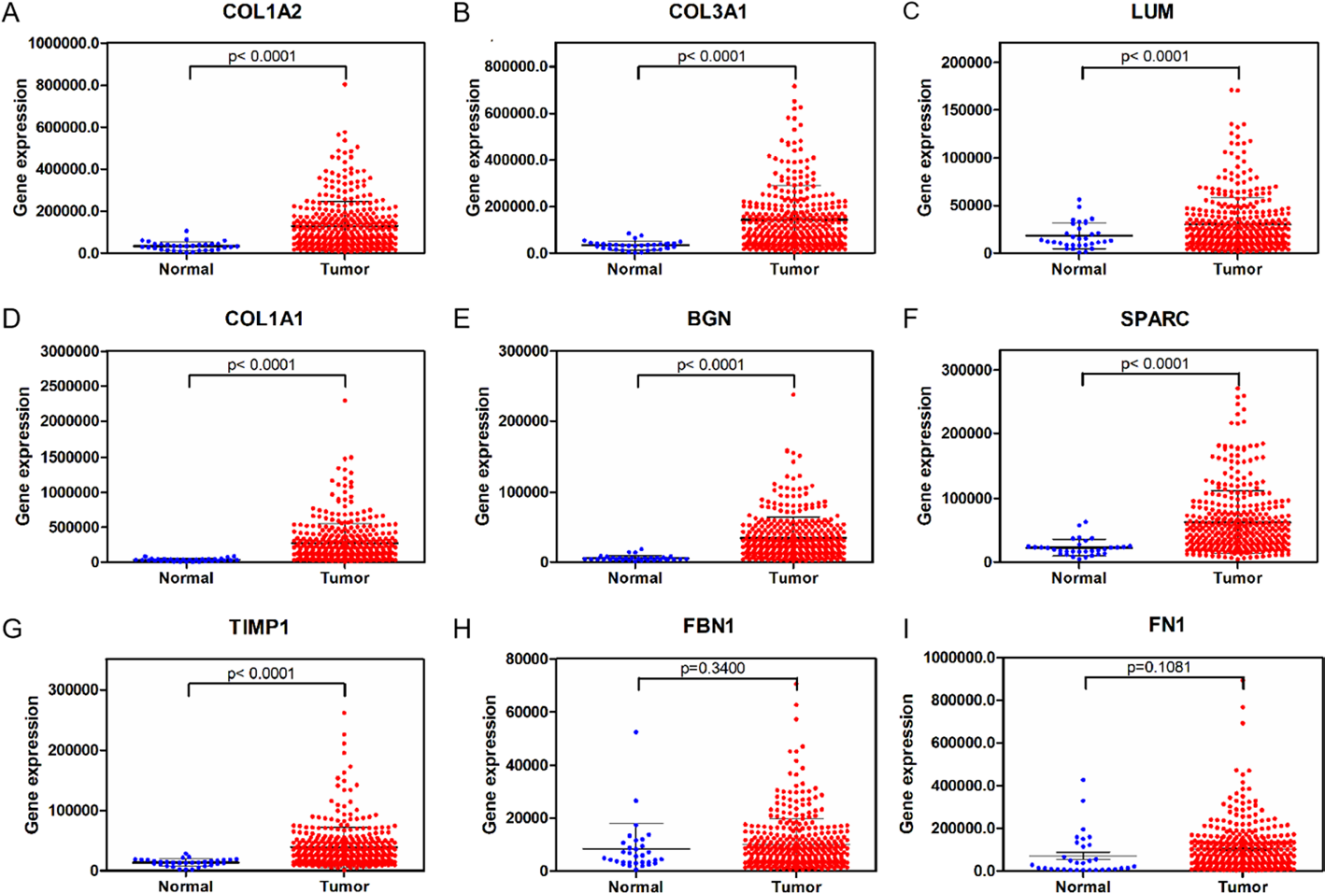
Validation of key genes of gastric cancer (GC) in the TCGA database. **A** Expression level of COL1A2 in GC and normal gastric tissues. **B** Expression level of COL3A1 in GC and normal gastric tissues. **C** Expression level of LUM in GC and normal gastric tissues. **D** Expression level of COL1A1 in GC and normal gastric tissues. **E** Expression level of BGN in GC and normal gastric tissues. **F** Expression level of SPARC in GC and normal gastric tissues. **G** Expression level of TIMP1 in GC and normal gastric tissues. **H** Expression level of FBN1 in GC and normal gastric tissues. **I** Expression level of FN1 in GC and normal gastric tissues

Through analysis of the TCGA database, we found that there were significant differences in the expression of COL1A2, COL3A1, LUM, COL1A1, BGN, SPARC and TIMP1 between cancer and normal tissues (*P* < 0.05) (Fig. 4A-I).

### Efficiency evaluation and verification of the risk model

**Fig. 5:**
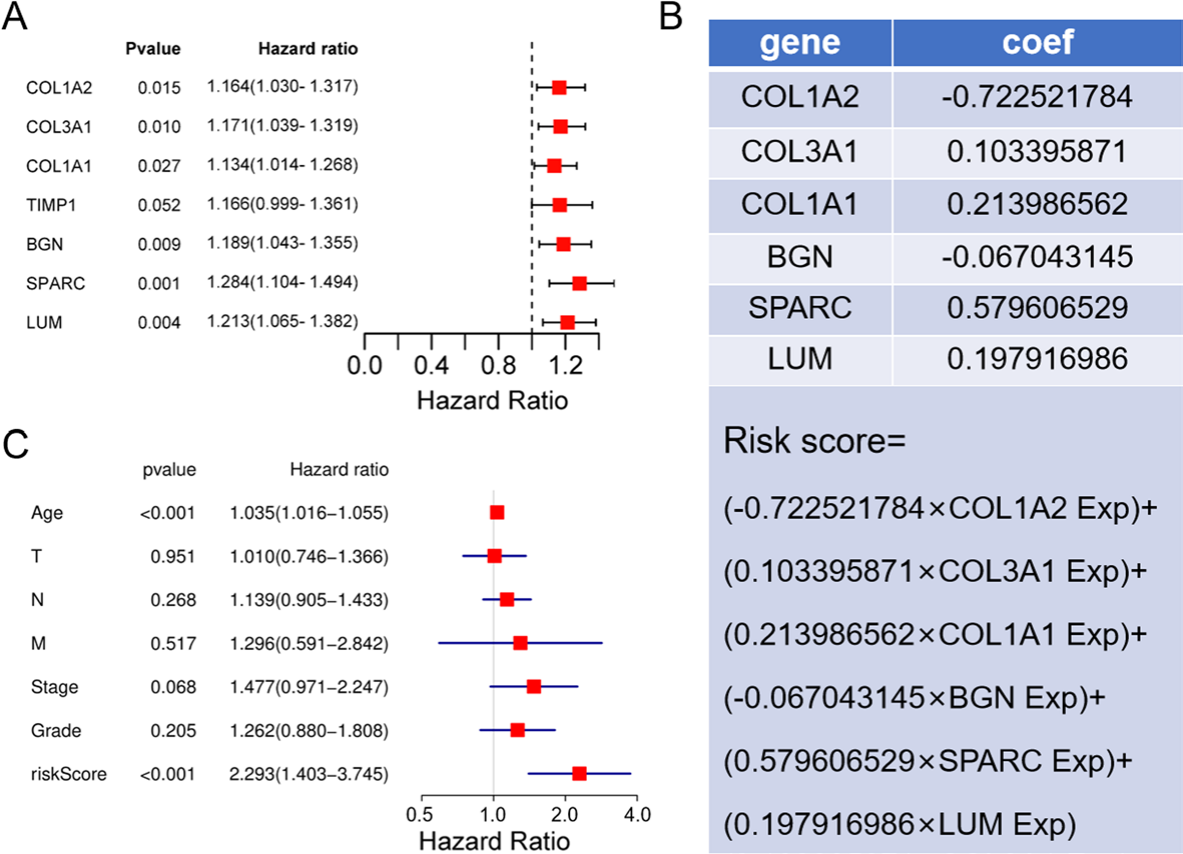
STAD patient risk model based on prognosis-related key genes. **A** Survival analysis was used to identify prognosis-related key genes. **B** Calculation formula of the comprehensive risk score of seven genes. **C** Multivariate independent prognostic analysis

Univariate Cox analysis of differentially expressed key genes showed that the expressions of COL1A2, COL3A1, COL1A1, LUM, BGN and SPARC were correlated with prognosis (Fig. 5A). Based on the key genes related to prognosis, a GC prognostic risk model was constructed. The calculation formula of the prognostic risk model was as follows: risk score = -0.722521784 × Expression(COL1A2) + 0.103395871 × Expression(COL3A1) + 0.213986562 × Expression(COL1A1)-0.067043145 × Expression(BGN) + 0.579606529 × Expression(SPARC) + 0.197916986 × Expression(LUM) (Fig. 5B).

**Fig. 6:**
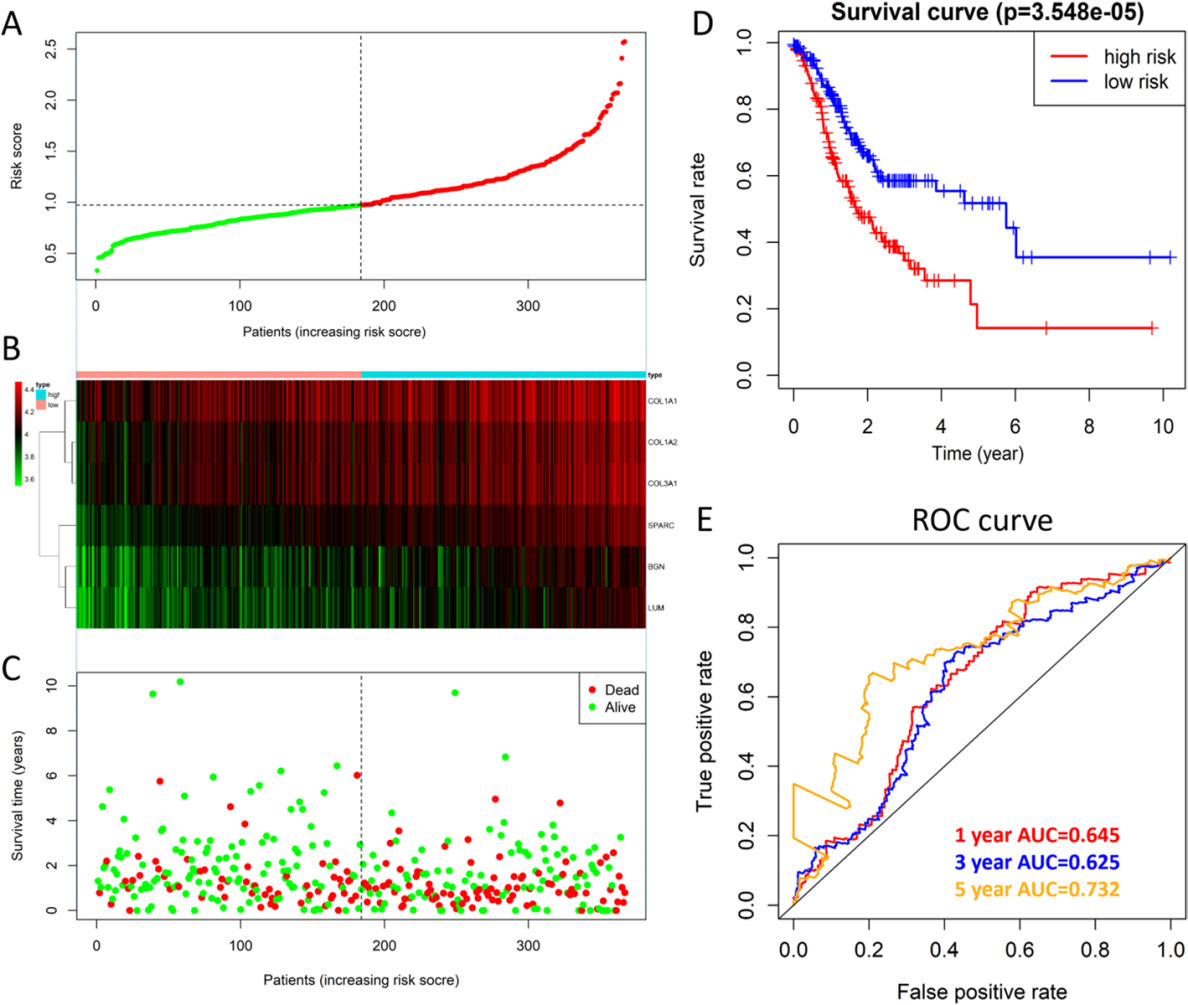
Performance evaluation of the prognostic risk scoring model. **A** Patients were sorted by risk score and grouped (red indicates high risk, and green indicates low risk). **B** Expression trend heatmap of the six model genes drawn with the "pheatmap" package in R software (Version 3.6.3, www.r-project.org) (blue represents the high-risk group, and red represents the low-risk group). **C** Distribution of survival status of patients in the high-risk group and the low-risk group (red indicates death, and green indicates survival). **D** KM survival curve of GC patients in high and low risk groups. **E** ROC curve of comprehensive risk score of six genes. The AUC (area under curve) indicates the area below the ROC curve. The value is between 0 and 1. The higher the value is, the better the prediction effect of the model

**Fig. 7:**
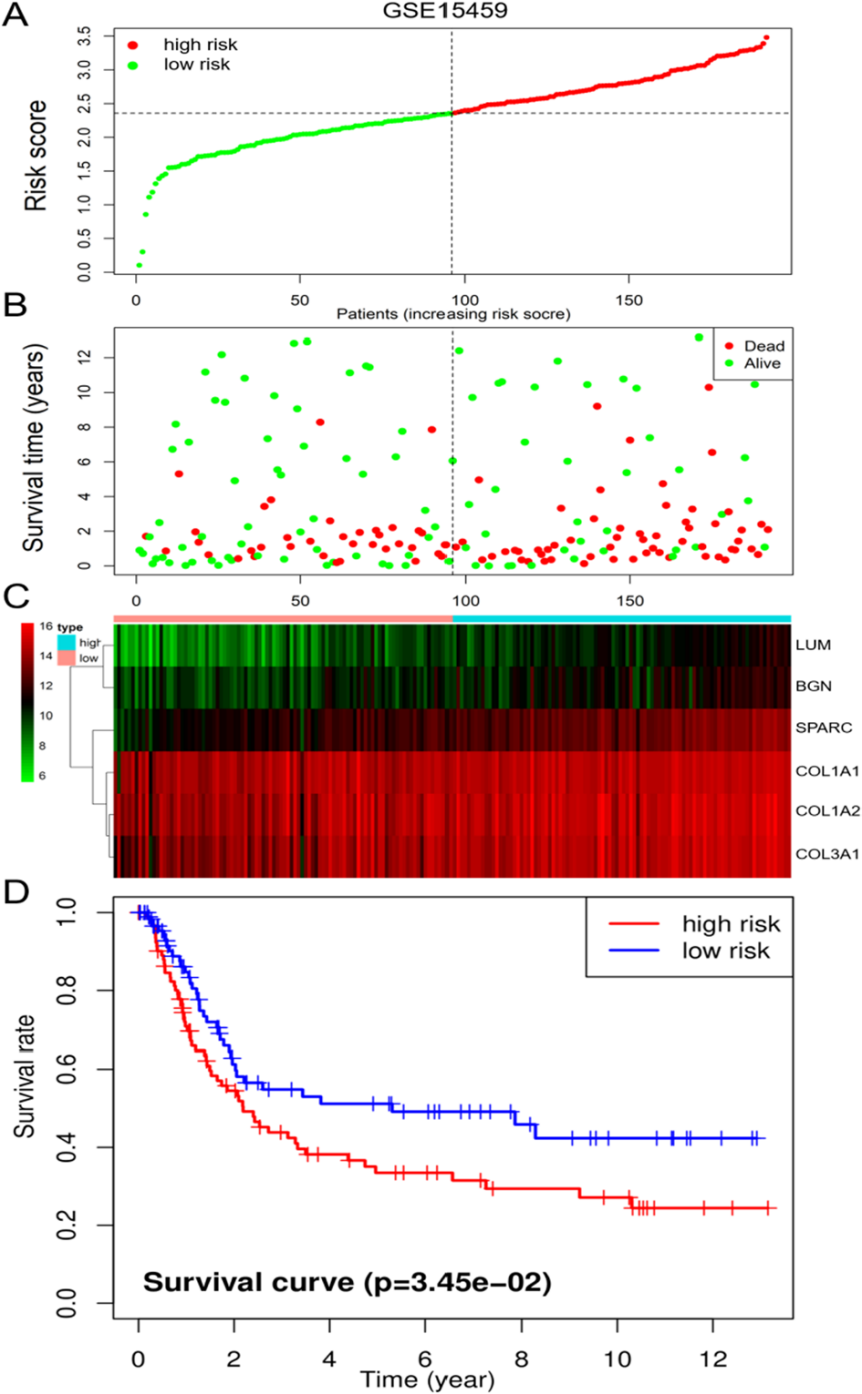
Validation of prognostic risk models in GEO databases. **A** Scatter plot of patient risk scores from low to high. Red dots represent the high-risk group. Blue dots represent the low-risk group. **B** Scatter plot distribution of survival time and survival status. Red represents death, and blue represents survival. **C** Expression trend heatmap of the six model genes drawn with the "pheatmap" package in R software (Version 3.6.3, www.r-project.org) (blue represents the high-risk group, and red represents the low-risk group). **D** Kaplan–Meier survival curves for OS in the high-risk and low-risk groups

**Fig. 8:**
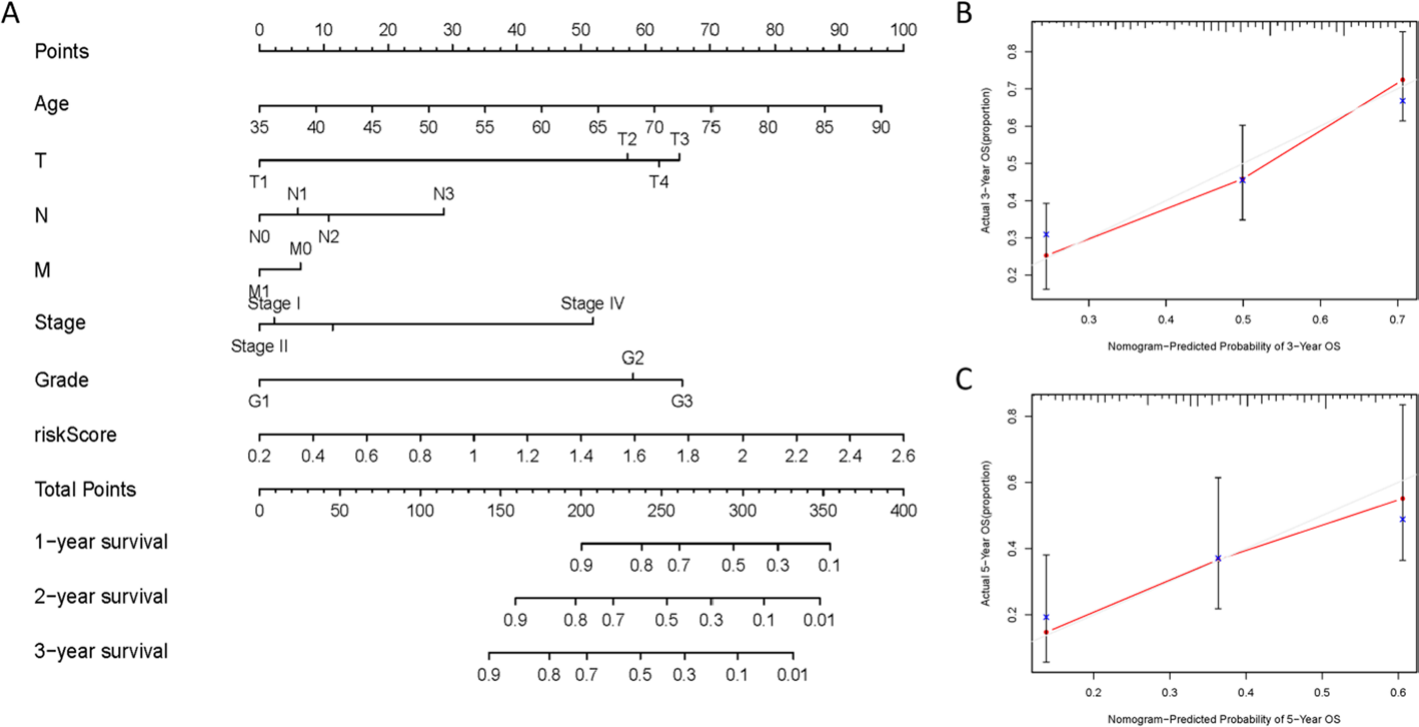
The nomogram can predict the 1-, 3- and 5-year overall survival of patients with STAD. **A** Nomogram of the prognostic model. **B**, **C** Calibration to test the accuracy of the constructed model to predict the 3- and 5-year survival status

**Fig. 9:**
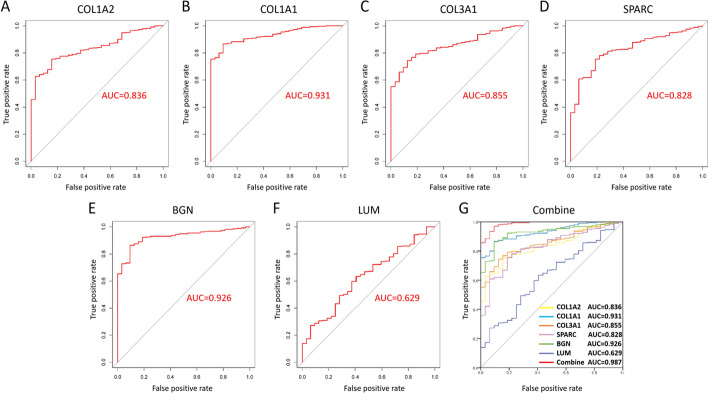
Receiver operating characteristic (ROC) curve analysis and area under the curve (AUC) statistics were used to evaluate the ability of prognosis-related key genes to distinguish gastric cancer (GC) from normal tissues. **A** COL1A2. **B** COL1A1. **C** COL3A1. **D** SPARC. **E** BGN. **F** LUM. **G** Combined

Multivariate Cox analysis showed that the risk score could be used as an independent prognostic factor for patients (Fig. 5C). Patients' risk scores were ranked, and the patients were divided into high- and low-risk groups (Fig. 6A). The results showed that an increase in the risk score was accompanied by increases in the expression levels of six genes (Fig. 6B), and the survival time of patients was shortened (Fig. 6C). The KM survival curve also confirmed that the survival rate of the high-risk group was significantly lower than that of the low-risk group (*P* = 3.548e-05, N = 40) (Fig. 6D). The results of the ROC curve analysis suggest that the risk model has a good ability to predict the 5-year survival rate of patients with gastric cancer (Fig. 6E). The risk score of STAD patients in the GSE15459 dataset was calculated according to the risk score formula. Patients were divided into a high-risk subgroup and a low-risk subgroup according to the median value (Fig. 7A). The distribution of survival status was similar to that of TCGA data, and death cases were denser in the high-risk subgroup (Fig. 7B). The heatmap shows the expression trend of the six model genes (Fig. 7C). The patients in the high-risk subgroup of the model had a poor prognosis(N = 192) (Fig. 7D). The nomogram prediction model showed that the risk score contributed the most to the prognosis (Fig. 8A). The calibration curve indicated that the predicted 3-year and 5-year survival values were basically consistent with the measured values, indicating that the nomogram prediction model has good consistency (Fig. 8B, C). In addition, the ability of prognostic genes to distinguish GC from normal tissues was analysed. The efficiency of the joint diagnosis by the six genes was the best, and the AUC value was 0.987 (Fig. 9).

### Construction of the model gene miRNA‒mRNA network

**Fig. 10:**
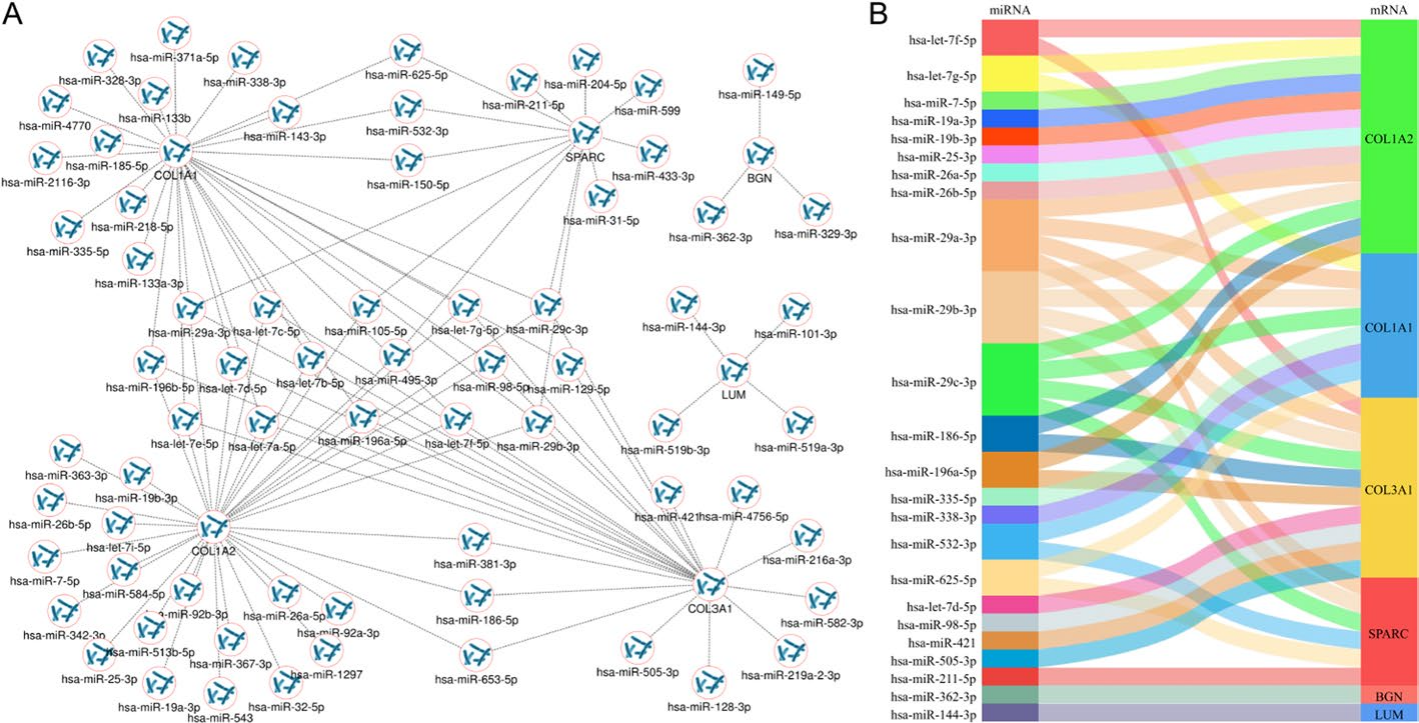
Construction of the miRNA‒mRNA regulatory network of the prediction model genes in the starBase database. **A** Model gene mRNA‒miRNA regulatory network. **B** Construction of miRNA‒mRNA regulatory network by mulberry map

We predicted the target miRNAs of the model mRNAs and found that 69 of them may bind to the model gene mRNAs (Fig. 10A). In addition, we predicted the correlations between mRNAs and miRNAs. Correlation analysis showed that the expression of 24 upstream miRNAs was negatively correlated with that of the model mRNAs (Supplementary data 1). Based on the above analysis results, we constructed the miRNA‒mRNA network of model genes (Fig. 10B). The number of upstream regulated miRNAs of COL1A2 was the largest, which made us interested in COL1A2. Therefore, COL1A2 was taken as the target gene for further analysis in this study.

### Expression of COL1A2 in tissues and cells

**Fig. 11:**
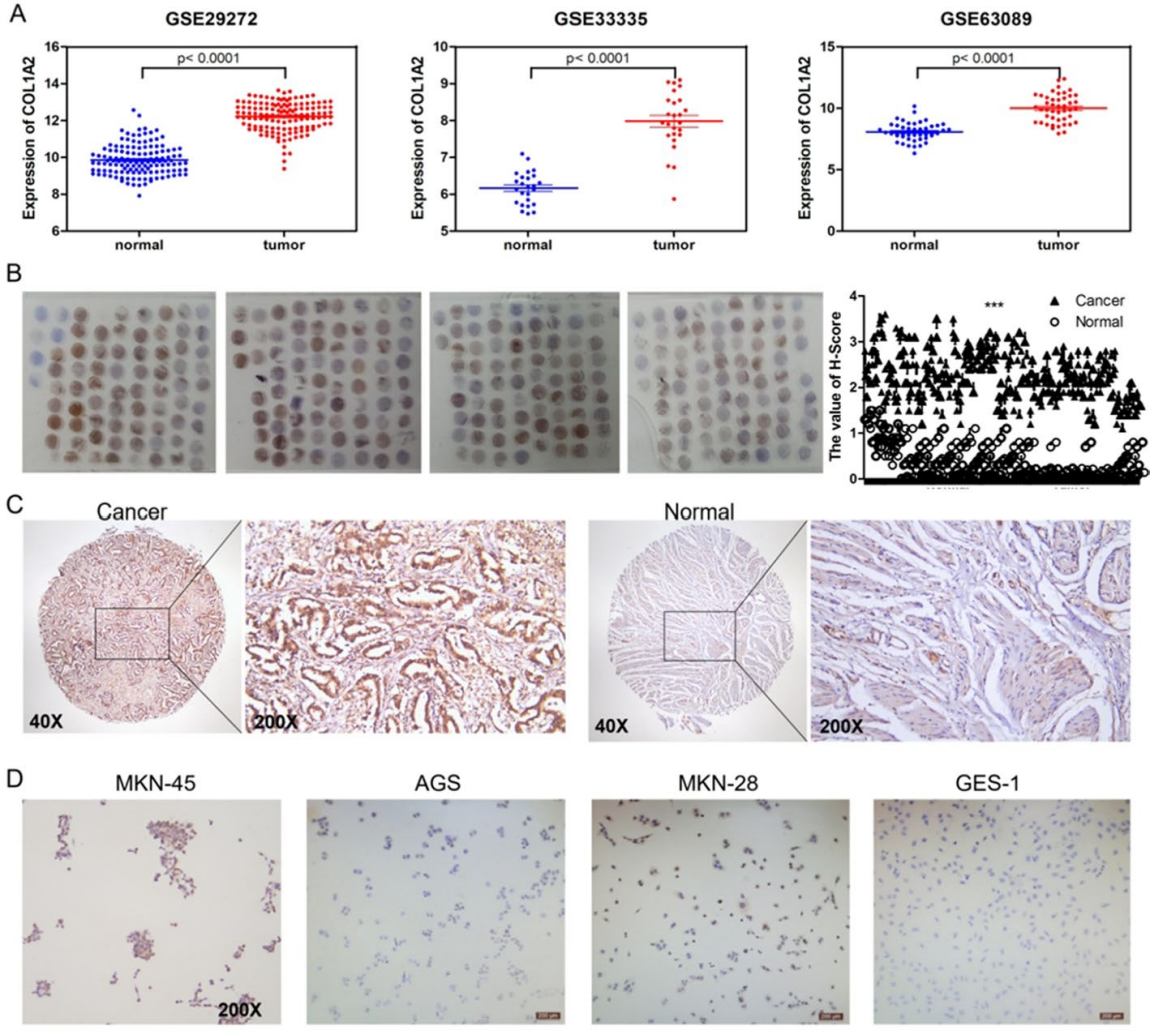
Expression of COL1A2 in tissues and cell lines. **A** In GSE29272, GSE33335 and GSE63089 microarray data, COL1A2 expression in cancer tissues was higher than that in normal tissues (*P* < 0.0001). **B** Expression level of COL1A2 in the tissue microarray. ***P < 0.001. **C** Immunohistochemical staining of COL1A2 in GC and adjacent tissues. **D** COL1A2 staining results in the MKN-45, MKN-28, AGS and GES-1 cell lines

In the GSE29272, GSE33335 and GSE63089 datasets, the expression of COL1A2 in cancer tissue was higher than that in normal tissue (*P* < 0.05) (Fig. 11A). In a microarray of 150 pairs of cancer and adjacent tissue, 144 pairs of cancer and adjacent tissue were included because of tissue shedding during the experiment. The expression of COL1A2 in gastric cancer tissues was higher than that in adjacent tissues (Fig. 11B, C). The correlation analysis between COL1A2 expression and clinical factors showed that high expression of COL1A2 was related to lymph node metastasis (Table 3). In the immunocytochemistry experiment, the brown granular region represented the region positive for COL1A2 protein expression. In the MKN-45 cell line, the nuclear and cytoplasmic regions showed different degrees of positivity, and the cytoplasmic signal was strong. In the MKN-28 cell line, the cytoplasmic region showed moderate positivity. In AGS cell lines, the cytoplasmic region showed weak positivity. In normal GES-1 epithelial cells, the nuclear and cytoplasmic regions were negative (Fig. 11D).

**Table 3:** Clinical characteristics of different patients in the COL1A2 high- and low-expression groups

Factors	COL1A2 expression		*P* value
	High	Low	
Sex			0.585
Male	52	49	
Female	20	23	
Age(years)			0.491
≥ 50	66	69	
< 50	6	3	
Tumour size (cm)			1.000
> 5	21	21	
≤ 5	51	51	
Lymph node metastasis			< 0.001
Positive	54	18	
Negative	18	54	
Differentiation			0.339
High	2	6	
Moderate	32	29	
Low	38	37	

## Discussion

The development of gastric cancer (GC) is a multistep process caused by several genetic alterations. The diagnosis, management, and prognosis of GC have not greatly improved because of a lack of extremely sensitive and noninvasive biomarkers [16]. Currently, bioinformatics analysis, which focuses on differentially expressed molecule screening, network-based core gene analysis, and survival analysis, has been widely employed to uncover possible biomarkers that can be used to improve the diagnosis, treatment, and prognosis of GC [17]. However, the capacity to discover possible biomarkers and diagnostic targets has been constrained because most studies have used only one sample or had small sample sizes. Numerous studies have been conducted by relevant academics on the pathogenesis of GC; however, the pathogenesis of GC is still not fully understood. Many genes are thought to be connected to the occurrence and development of GC. Finding early diagnostic markers and the main therapeutic targets for GC is crucial, but research in this area is still in its infancy. We screened and downloaded three typical GC datasets from the GEO database for thorough analysis in this work to pinpoint the critical genes associated with the prognosis of GC patients.

From the GSE29272, GSE33335, and GSE63089 datasets, 106 overlapping DEGs, including 67 upregulated genes and 39 downregulated genes, were screened out. Extracellular matrix–related terms, including collagen, extracellular matrix structural constituents, extracellular matrix organization, and extracellular structure organization, were associated with the key gene cluster in which the upregulated genes were focused, according to GO enrichment analysis. The upregulated DEGs were primarily enriched in ECM-receptor interaction, focal adhesion, protein digestion and absorption, the PI3K-Akt signalling pathway, and leukocyte transendothelial migration, according to KEGG pathway analysis. Notably, the extracellular matrix (ECM) was present in most DEG enrichment outcomes. The ECM is an intricate network structure that provides cells with basic metabolic and structural support. ECM is altered during the carcinogenesis process. The primary elements of the ECM are collagen, laminin, and fibronectin, and together, they create a microenvironment in which cancer cells can develop, endure, and migrate. All stages of the disease, from the initial tumour formation to metastasis, have been shown to involve the ECM in GC [18]. The PI3K-Akt signalling pathway is crucial for controlling apoptosis, protein synthesis, and cell growth [19]. The adhesion signalling pathway is involved in tumour growth and metastasis as well as many other critical biological processes, including cell adhesion signalling, cytoskeleton rearrangement, cell survival, and apoptosis [20]. Two gene clusters that were the most closely associated and were situated in significant nodes were chosen after the PPI network made of various genes was analysed using the MCODE plug-in of Cytoscape software. Each gene cluster made up a comparatively independent network, and the genes therein may perform related biological tasks through antagonistic or synergistic interactions.

In this study, nine core genes (COL3A1, FN1, BGN, SPARC, COL1A1, COL1A2, FBN1, LUM and TIMP1) were identified by constructing a protein interaction network. Through further analysis of the TCGA database, it was found that there were significant differences in the expression of seven genes (COL3A1, BGN, SPARC, COL1A1, COL1A2, LUM and TIMP1). However, six of the above seven genes (COL3A1, BGN, SPARC, COL1A1, COL1A2 and LUM) were associated with the prognosis of GC. Based on the above prognostic genes, a risk model was constructed, and a nomogram was established to predict the prognosis of patients. The calibration curve shows that the survival prediction of the nomogram was in good agreement with the actual survival. Through validation of the GSE21653 dataset, it was found that the model has certain value for predicting the clinical prognosis of GC patients. Relevant studies have found that miRNAs participate in a variety of biological behaviours, such as cell proliferation, apoptosis and invasion, by regulating the expression of target genes [21]. The miRNA‒mRNA regulatory mechanism is widespread in cancer. Therefore, we explored the mechanism of miRNA regulation of model genes. We screened 24 upstream miRNAs suitable for the ceRNA hypothesis and constructed a miRNA network of model genes. The number of upstream regulatory miRNAs of COL1A2 was the largest. Among them, hsa-miR-7-5p [22], hsa-miR-19b-3p [23], hsa-miR-25-3p [24], hsa-miR-26a-5p [25], hsa-miR-26b-5p [26], hsa-miR-29a-3p [27], hsa-miR-29b-3p [27], hsa-miR-29c-3p [28], and hsa-miR-196a-5p [29] have been reported to be closely related to GC. However, the regulatory mechanisms of upstream miRNAs and COL1A2 need further exploration and analysis.

Type I collagen, which makes up the majority of the ECM, is represented by COL1A2. In pancreatic cancer, COL1A2 is abundantly expressed and has been shown to encourage tumour cell growth and migration [30]. According to a previous publication, the transcription factor TBX3 controls COL1A2, and activation of COL1A2 encourages chondrosarcoma migration [31]. Activation of COL1A2 can make gastric cancer resistant to Apatinib [32] and promote proliferation and invasion of gastric cancer cell [33]. We analysed the expression of COL1A2 in GC tissues and cell lines. The results showed that the expression of COL1A2 in GC tissue was significantly higher than that in adjacent tissue. In the gastric cancer cell lines MKN-45, MKN-28 and AGS, the expression of COL1A2 was stronger than that in the normal gastric epithelial cell line GES-1. In addition, we investigated the relationships between COL1A2 expression and clinical parameters. It was found that COL1A2 expression was associated with lymph node metastasis of GC. This result confirmed that the high expression of COL1A2 was associated with poor prognosis and COL1A2 may mediate malignant progression of gastric cancer metastasis and transformation through lymph node metastasis. Therefore, COL1A2 may be a reliable prognostic index and therapeutic target of GC.

Regarding the study's shortcomings, it should be noted that no molecular mechanisms were investigated in vitro or in vivo; instead, the study relied on database analysis and clinical patient COL1A2 immunohistochemistry results. The selection of datasets has limitations and may lead to bias in the analysis results. The value of the key genes can be further explored from the perspective of clinical molecular pathology to improve diagnosis and targeted therapy.

## Conclusion

In conclusion, 9 core genes of GC were discovered using bioinformatics tools to filter and assess the differentially expressed genes involved in the regulation of GC. On the basis of prognosis-associated genes, a prognostic risk model was further examined and developed. The nomogram developed using the risk model can accurately estimate the survival rate of patients, assist physicians in developing individualized prognostic assessments, and determine the best course of therapy for each patient. A novel prognostic marker for GC may exist given the result of the validation experiment, which demonstrated that COL1A2 is highly expressed in GC and associated with poor patient prognoses. This study also offers novel prognostic indicators and targets for the diagnosis and treatment of GC as well as new candidate genes and a theoretical framework for the study of targeted therapy for GC. Nevertheless, the pathogenic mechanisms of these genes in GC still need to be confirmed by a large number of basic experiments and clinical studies.

## Supplementary Information

Supplementary Material 1.

## Data Availability

Publicly available datasets were analyzed in this study. This data can be found here: GEO data base, accession number: GSE29272, GSE33335, GSE63089 and GSE15459( http://www.ncbi.nlm.nih.gov/geo/). TCGA data base website (https://portal.gdc.cancer.gov/). Protein protein interaction (PPI) network analysis data are available in the STRING database (https://www.string-db.org/). The prediction data of miRNAmRNA network is from starBase database (https://starbase.sysu.edu.cn/).

## Abbreviations

GC: Gastric cancer
GEO: Gene expression omnibus
DEGs: Differentially expressed genes
GO: Gene ontology
KEGG: Kyoto encyclopedia of genes and genomes
FC: Fold change
PPI: Protein‒protein interaction
MCODE: Molecular complex detection
MCC: Maximal clique centrality
ROC: Receiver operating characteristic
AUC: Area under the curve
MF: Molecular function
CC: Cell components
BP: Biological process
COL1A2: Collagen alpha-2 (I) chain
SPARC: Secret protein acid and rich in cystine
LUM: Lumican
FBS: Foetal bovine serum
AUC: Area under the curve
ECM: Extracellular matrix
IHC: Immunohistochemistry
STAD: Stomach adenocarcinoma
